# Comparison of operative and non-operative management of fifth metatarsal base fracture: A meta-analysis

**DOI:** 10.1371/journal.pone.0237151

**Published:** 2020-08-13

**Authors:** Yanming Wang, Xu Gan, Kai Li, Tao Ma, Yongxiang Zhang

**Affiliations:** Zaozhuang Hospital of Traditional Chinese Medicine, Zaozhuang, Shandong, China; Assiut University Faculty of Medicine, EGYPT

## Abstract

Fracture to fifth metatarsal’s base is one the most common injury experienced at the foot. Studies have for long debated the use of operative and non-operative interventions for the management of the fracture, especially owing to its peculiar vasculature. However, to date, no attempt has been made to synthesize the evidence comparing the efficacy of operative and non-operative interventions for managing the fifth metatarsal’s base fracture. To meta-statistically compare the effects of operative and non-operative management of fifth metatarsal base fracture. A systematic identification of literature was performed according to PRISMA guidelines on four academic databases: MEDLINE, Scopus, EMBASE, and CENTRAL. A meta-analysis evaluated the influence of operative and non-operative interventions on rate of non-union, mean duration of union, duration of return to activity, duration of return to sport, visual analog scale, and the American orthopedic foot & ankle scale. Out of 1,170 records, 11 articles including 404 participants (mean age: 29.8 ± 7.4 years) were included in this review. This systematic review presents a 1b level of evidence supporting the use of operative interventions for enhancing fracture union as compared to non-operative interventions. The meta-analysis reveals beneficial effects for operative interventions by demonstrating *medium to large* effect reduction of rate of non-union (Hedge’s g: -0.66), duration of union (-1.7), duration of return to activity (-2.07), visual analog scale (-0.86), and enhancement of the American orthopedic foot & ankle scale score (0.73) as compared to non-operative intervention. The current systematic review and meta-analysis recommend the use of operative interventions for managing the fifth metatarsal’s base fracture. The review reports beneficial effects of operative interventions as compared to non-operative interventions for reducing the rate of non-union, duration of union, duration of return to activity, duration of return to sport, visual analog scale, and increasing the American orthopedic foot & ankle scale score.

## Introduction

Fracture of the fifth metatarsal’s base is one of the most common stress fractures encountered in the lower extremities [1–4]. The fracture is characterized by a transverse disruption at the diaphyseal and metaphyseal junction of the proximal 1/3^rd^ of the metatarsal bone [5]. According to the recent epidemiological studies, this fracture accounts for almost 40–75% of all fractures encountered at the foot [6, 7]. Moreover, studies report that the onset of this fracture at the fifth metatarsal’s base is highly prevalent in both sports [8, 9], and sedentary settings [10, 11].

Literature suggests many reasons which could be behind the higher prevalence of this fracture across different population groups [12]. Firstly, the biomechanical insufficiency of the fifth metatarsal during inverse axial-loading at the ankle joint has been suggested to be a predominant reason predisposing towards this stress fracture [13, 14]. Secondly, a higher correlation between metatarsal fractures and lower levels of bone mineral density as in osteoporotic [13], and post-menopausal women [14], is an additional reason due to which this fracture is common in senile population groups [3, 6].

Typically, the management of the fifth metatarsal’s base fracture has been pursued by either operative [15], or non-operative measures [16, 17]. The operative measures usually include an internal fixation approach with an intramedullary, bicortical screw and/or bone graft-inlay [18, 19]. Whereas, the non-operative measure employs an immobilization cast aimed to facilitate passive healing with/without weight-bearing [17, 20]. The choice of strategy to be utilized, however, is usually inclined upon the various classifications of fracture reported in the literature [19, 21–23]. The most widely used of them, the Torg classification, distinguishes the fracture in three different subtypes based on their healing potential [16, 19]. The classification suggests that the presence/absence of medullary sclerosis on the fracture margins could substantially influence the prognostic outcomes associated with the choice of intervention [19]. Besides, another important aspect that requires due diligence on the behalf of the clinician for selecting an appropriate intervention is the vasculature of the fracture site [24, 25]. According to Smith et al. (1992), the presence of an avascular zone in the proximal-diaphysis after a fracture (disrupted supply from the nutrient artery) could affect the decision-making process concerning the choice of intervention because of its influence on the prognostic outcome of the fracture.

Despite the advancements in the past decades concerning development of various interventions [26–28], and anatomical specifications [29, 30], a consensus concerning an optimal choice of intervention for managing fifth metatarsal’s base fracture is still missing [20]. While on one hand, a part of the literature recommends the aggressive use of operative intervention because of their ability to enhance the rate of fracture union, duration of union, and duration of return to sports [9, 31]. On the other hand, a part of literature recommends the use of non-operative interventions to facilitate recovery [15, 16]. The studies suggest that the use of non-operative interventions can avoid the complications, discomfort associated with the surgery and that too in a cost-effective manner [16, 32]. In addition to that, the recent systematic reviews too provide inconclusive evidence regarding the optimal choice of treatment [16, 33, 34]. Taken together, this lack of consensus has proven to be a challenging avenue for the clinicians to develop an efficient decision-making model for selecting optimal interventions for managing the fifth metatarsal’s base fracture [15].

Therefore, this present study aims to address this gap in the literature by synthesizing the current state of evidence concerning operative and non-operative interventions to manage the fifth metatarsal’s base fracture. This review will provide comprehensive evidence concerning the rate of non-union, duration of union, duration of return to activity, visual analog scale, and the American orthopedic foot & ankle scale score between operative and non-operative interventions.

## Methods

This systematic review and meta-analysis was carried in adherence to PRISMA guidelines [35]. A PRISMA checklist has been provided in the S1 File

### Data search strategy

We searched four academic databases (MEDLINE, CENTRAL, EMBASE and Scopus) from inception until December 2019 using MeSH keywords: “Jones fracture”, “metatarsal fracture”, “fifth metatarsal fracture”, “fifth metatarsal base fracture”, “5^th^ metatarsal fracture”, “V metatarsal fracture”, “base of fifth metatarsal fracture”, “fracture”, “open fracture reduction”, “operation”, “internal fixation”, “splint”, “plaster cast”, “conservative”. In addition, we screened the bibliography of the included studies for any additional relevant study. The inclusion criteria for the included studies were as follows:

- Studies compared the efficacy of operative and non-operative approaches on the healing of fifth metatarsal base fracture in humans.
- Studies evaluated the outcome of fracture union (e.g. rate of non-union, duration of reunion, duration of return to normal activity, sport, visual analog scale, American orthopedic foot & ankle scale, EQ-5D, etc.).
- Studies were either randomized controlled trials, quasi-randomized controlled trials, controlled clinical trials, prospective observational trials with control groups or retrospective trials.
- Studies published in peer-reviewed scientific journals, conferences.
- Studies published in the English language.

In terms of the exclusion criteria, we excluded studies that evaluated the efficacy of operative and non-operative interventions on fractures other than that of the fifth metatarsal base. We excluded unpublished grey literature on the basis of the fact that they score poorly on methodological quality, are less likely to conceal information regarding the allocation of participants and blinding [36]. We also excluded studies that were not published in English language. The selection procedure was independently replicated by two reviewers to avoid biasing. The following data was extracted from the included studies: authors, sample description (gender, age), method of assessment, intervention, follow-up duration, and outcome measures. In the articles where quantitative data outcomes were incomplete or not mentioned the reviewers made attempts to contact respective corresponding authors for additional data.

### Quality assessment

The risk of bias in the included studies was assessed by Cochrane risk of bias assessment tool for randomized controlled trials and non-randomized controlled trials i.e. ROBINS-I [37, 38]. The included studies were independently appraised by two reviewers. Inadequate randomization, concealment of allocation and reporting of selective outcomes were considered as major threats for biasing [39]. In cases of ambiguity, discussions were held between the reviewers until a consensus was reached. Moreover, a level of evidence analysis based on the center for evidence-based medicine was also included [40].

### Data analysis

A within-group meta-analysis of the included studies was carried out using CMA (Comprehensive Meta-analysis version 2.0) [41]. The data was distributed and separately analyzed for the rate of non-union, duration of reunion, duration of return to normal activity, American orthopedic foot & ankle scale, and visual analog scale scores. A meta-analysis was conducted based on the random-effects model [42]. The effect sizes are reported as weighted Hedge’s g. The thresholds for interpreting the weighted effect sizes are: ≤ 0.2 a *small* effect, ≤ 0.5 as a *medium* effect and ≥ 0.8 a *large* effect [43]. Heterogeneity was assessed by computing I^2^ statistics. The thresholds for interpreting heterogeneity are: 0–25% with negligible heterogeneity, 25%-75% with moderate heterogeneity and ≥75% with substantial heterogeneity [44]. Sensitivity analyses were performed in cases where substantial sources of heterogeneity existed [45]. Here, based on the presence or absence of inadequate randomization methods in the studies we either included or excluded the results of the studies. For each evaluated parameter details of weighted effect size, 95% confidence intervals, level of significance and heterogeneity have been duly reported. Besides, we analyzed publication bias by performing Duval and Tweedie's trim and fill procedure [46]. This non-parametric method estimates the number of missing studies that might exist and the effects they might have on the outcome of a meta-analysis. Here, asymmetric studies are imputed from the left side of the plotted graph to identify the unbiased effect. Thereafter, these trimmed effects are refilled in the plotted graph and then the combined effect is recalculated. In the present review, the alpha level was set at 5%.

## Results

**Fig 1:**
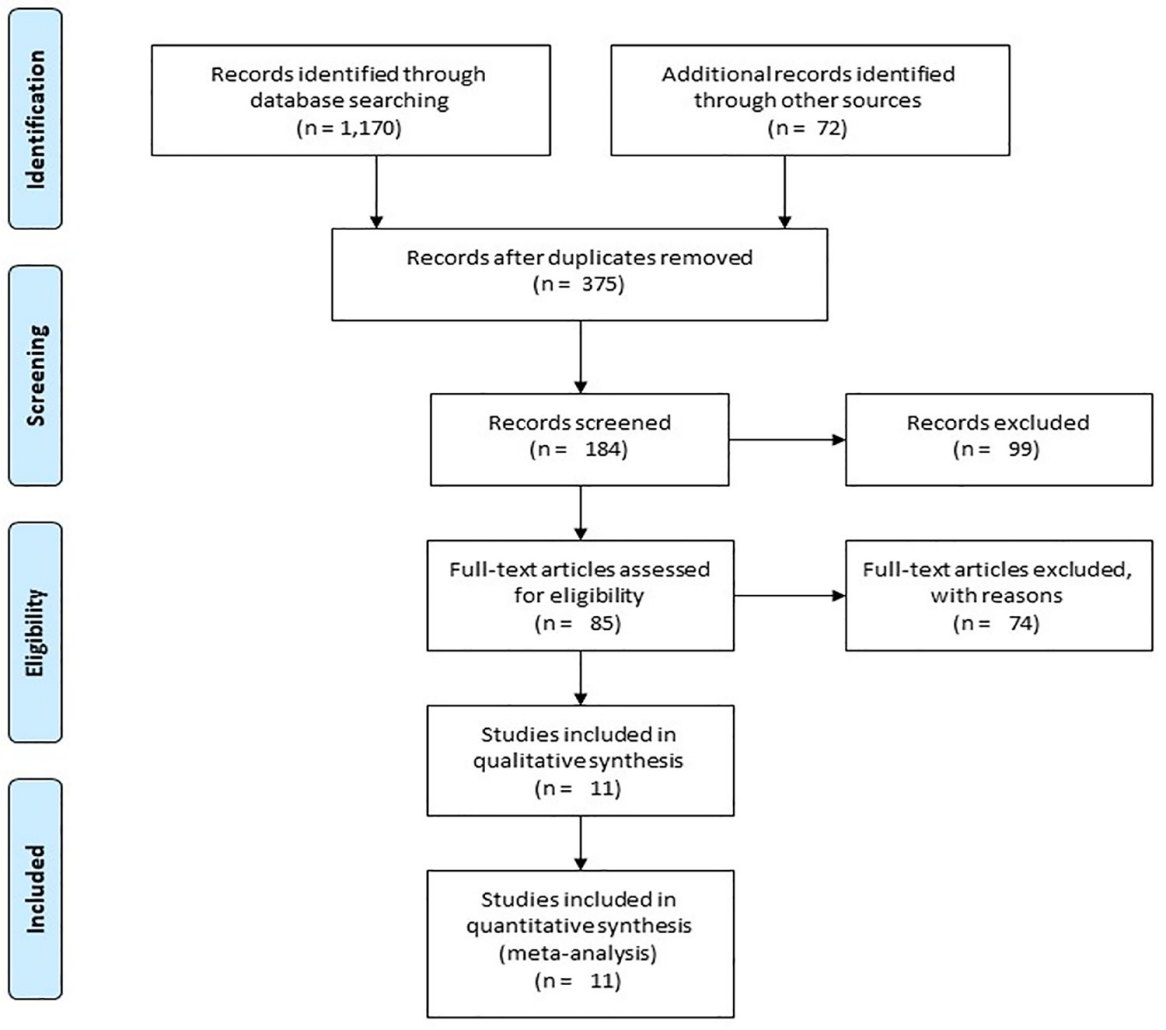
Illustrates the PRISMA flow chart for the included studies.

A preliminary search on four academic databases resulted in a total of 1,170 studies, 72 more studies were included after the bibliography of these articles were screened (Fig 1). Thereafter, upon excluding the duplicates and applying the inclusion criteria, a total of 11 studies were retained. In the included studies, four were randomized controlled trials [10, 47–49], whereas seven were non-randomized retrospective trials [2, 50–55].

Nine studies analyzed the influence of operative and non-operative interventions on the rate of non-union of the fifth metatarsal base fracture [2, 10, 47–52, 54]. Here, five studies reported significant (p<0.05) reduction in rate of non-union[2, 47, 48, 51, 52], and three reported non-significant (p>0.05) reduction for the operated group as compared to non-operated group [10, 49, 54]. One study reported no difference between the operated and non-operated group [50]. Eight studies evaluated the mean duration of union [10, 48–50, 52–55]. Here, four studies reported significant reduction in the duration of union [10, 48, 49, 52], and three studies reported non-significant reduction for the operated group as compared to non-operated group [53–55]. One study reported no difference between the operated and non-operated group [50]. Three studies evaluated the mean duration of return to sport [48, 50, 51], and two studies evaluated the mean duration of return to normal activities of daily living [10, 52]. Two studies each reported a significant reduction in duration of return to normal activity [10, 52], and return to sport [48, 50], for the operated group as compared to the non-operated group. One study reported a non-significant reduction in the duration of return to sport for the non-operated group as compared to the operated group [51]. Likewise, five studies each evaluated visual analog scale score [10, 49, 53–55], and American orthopedic & foot scale score [47, 49, 53–55]. For the visual analog scale score, two studies each reported significant [10, 49], and non-significant [54, 55], reduction in the visual analog scale scores for the operated group as compared to non-operated group. One study reported no differences between the operated and non-operated group [53]. Similarly, regarding the American orthopedic & foot scale assessment, two studies each reported significant [47, 55], and non-significant [49, 54], reduction in the American orthopedic & foot scale score for the operated group as compared to non-operated group. One study reported no differences between the operated and non-operated group [53]. A detailed qualitative description of all the studies has been summarized in Table 1.

**Table 1:** Illustrates the characteristics of the included studies.

Author	Age: M ± S.D years	Sample size	Assessment	Intervention	Follow-up (months)	Outcome
Demel et al. (2019)	O: 25.5 ± 6.9	O: 15	American orthopedic foot & ankle society scale and rate of non-union	O: Fixation with Herbert-type headless two threaded bolt and bandage	3	Significant enhancement in American orthopedic foot & ankle society scale score in O as compared to N-op. Significant reduction in rate of non-union in O as compared to N-op.
	N-op: 28.7 ± 7.5	N-op: 12				
				N-op: Plaster cast		
Park et al. (2017)	O: 47.2	O: 13F, 9M	Rate of non-union, duration of union, American orthopedic foot & ankle society scale score & visual analog scale	O: Internal fixation with intramedullary screw and plaster cast	6	Reduction in visual analog scale score, duration of union and rate on non-union in O as compared to N-op. Higher American orthopedic foot & ankle society scale score in O as compared to N-op.
	N-op: 38.8	N-op: 14F, 10M				
				N-op: Plaster cast		
Wu et al. (2017)	O: 25.5 ± 6.9	O: 8F, 13M	Visual analog scale, American orthopedic foot & ankle society scale, rate of non-union and recovery duration	O: Fixation with percutaneous screw fixation	12	Significant reduction in visual analog scale, duration of union, in O as compared to N-op. Enhancement in American orthopedic foot & ankle scale score in O as compared to N-op. Higher rate of non-union in N-op as compared to O.
		N-op: 7F, 13M				
	N-op: 28.7 ± 7.5					
				N-op: Plaster cast		

Lee et al. (2016)	14–73	16F, 13M	Visual analog scale, American orthopedic foot & ankle society scale and duration of union	O: Open reduction & internal fixation	2	Reduced duration of union in O as compared to N-op. No difference in visual analog scale and American orthopedic foot & ankle society scale scores in between O and N-op.
	O: -	O: 9				
	N-op: -	N-op: 20				
				N-op: Plaster cast		
Sokkar and Abdelkafy (2016)	O: 28.7 ± 8.8	O: 12M	Visual analog scale, American orthopedic foot & ankle society scale and duration of union	O: Fixation with bicortical cancellous screw	6	Significant enhancement in American orthopedic foot & ankle society scale score in O as compared to N-op. Reduction in visual analog scale, duration of union, in O as compared to N-op.
		N-op: 12M				
	N-op: 29.5 ± 7.9					
				N-op: Plaster cast		
Ekstrand and van Dijk (2013)	18–33	O: 28M	Rate of non-union and duration of return to sport	O: Internal fixation with intramedullary screw	-	Significantly reduced rate of non-union reported for O as compared to N-op. Reduced healing duration in N-op as compared to O.
	O: -	N-op: 9M				
	N-op: -					
				N-op: -		
Adhikari and Thakur (2010)	O: -	O: 7F, 8M	Rate of non-union, duration of union, visual analog scale score and duration of return to normal activity	O: Internal fixation with intramedullary screw and plaster cast	12	Significant reduction in visual analog scale, duration of union and duration of return to normal activity in O as compared to N-op. Reduced rate of non-union in O as compared to N-op.
		N-op: 8F, 8M				
	N-op: -					
				N-op: Plaster cast		

Chuckpaiwong et al. (2008)	27 ± 11.1	8F, 24M	Rate of non-union, duration of union and duration of return to sport	O: Internal fixation with intramedullary screw and plaster cast	40	Significant reduction in the duration of return to sport for O as compared to N-op. No differences in rate of non-union and duration for union between O and N-op.
	O: -	O: 18				
	N-op: -	N-op: 17				
				N-op: Plaster cast		
Mologne et al. (2005)	18–58	2F, 35M	Rate of non-union, duration of union and duration of return to sport	O: Internal fixation with intramedullary screw and non-weight bearing splint	25.3	Significant reduction in duration of union, rate of non-union and duration of return to sport for O as compared to N-op.
		O: 19				
	O: -					
	N-op: -	N-op: 18				
				N-op: Plaster cast		
Josefsson et al. (1994)	17–74	8F, 55M	Rate of non-union	O: Internal fixation with intramedullary screw and elastic bandage, cast	60	Significantly reduced rate of non-union for O as compared to N-op.
	O: -	O: 22				
		N-op: 44				
	N-op: -					
				N-op: Plaster cast		
Kavanaugh et al. (1978)	15–42	O: 13	Rate of non-union, duration of union and duration of return to normal activity	O: Internal fixation with intramedullary screw and plaster cast	42	Significant reduction in duration of union, rate of non-union and duration of return to normal activity in O as compared to N-op.
	O: -	N-op: 18				
	N-op: -					
				N-op: Plaster cast		


O: Operated group, N-op: Non-operated group

### Risk of bias

#### Randomized controlled trials

**Fig 2:**
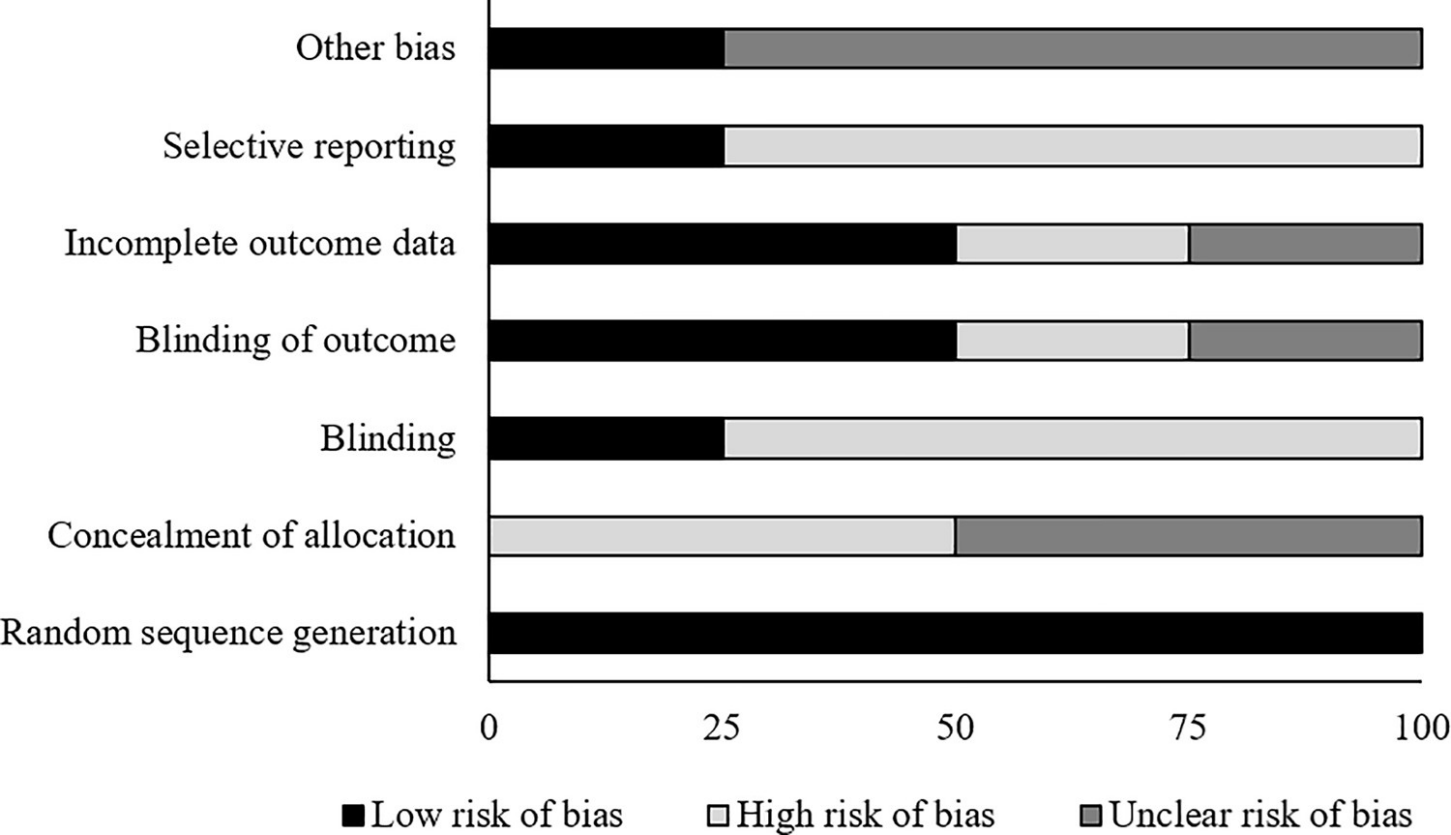
Illustrates the risk of bias (%) within studies according to Cochrane risk of bias assessment tool for randomized controlled trials.

The risk of bias for the randomized controlled trials according to Cochrane’s risk of bias assessment tool for randomized controlled trials has been demonstrated in Table 2. The overall risk in the included studies is poor. The highest risk of bias was observed to be due to lack of blinding of the participants, selective reporting and concealment of allocation Fig 2. A level of evidence of 1b was observed for all the included studies based on their experimental design.

**Table 2:** Illustrates the quality of the analyzed studies according to the Cochrane risk of bias assessment tool for randomized controlled trials.

Study	Random sequence generation	Concealment of allocation	Blinding	Blinding of outcome	Incomplete outcome data	Selective reporting	Other bias	Level of evidence
Mologne et al. (2005)	**+**	**-**	**-**	**+**	**-**	**-**	**?**	1b
Adhikari and Thakur (2010)	**+**	**?**	**-**	**?**	**+**	**-**	**+**	1b
Demel et al. (2019)	**+**	**-**	**-**	**-**	**?**	**-**	**?**	1b
Wu et al. (2017)	**+**	**?**	**+**	**+**	**+**	**+**	**?**	1b

-: high risk of bias, +: low risk of bias,?: unclear risk of bias

#### Controlled clinical trials

**Fig 3:**
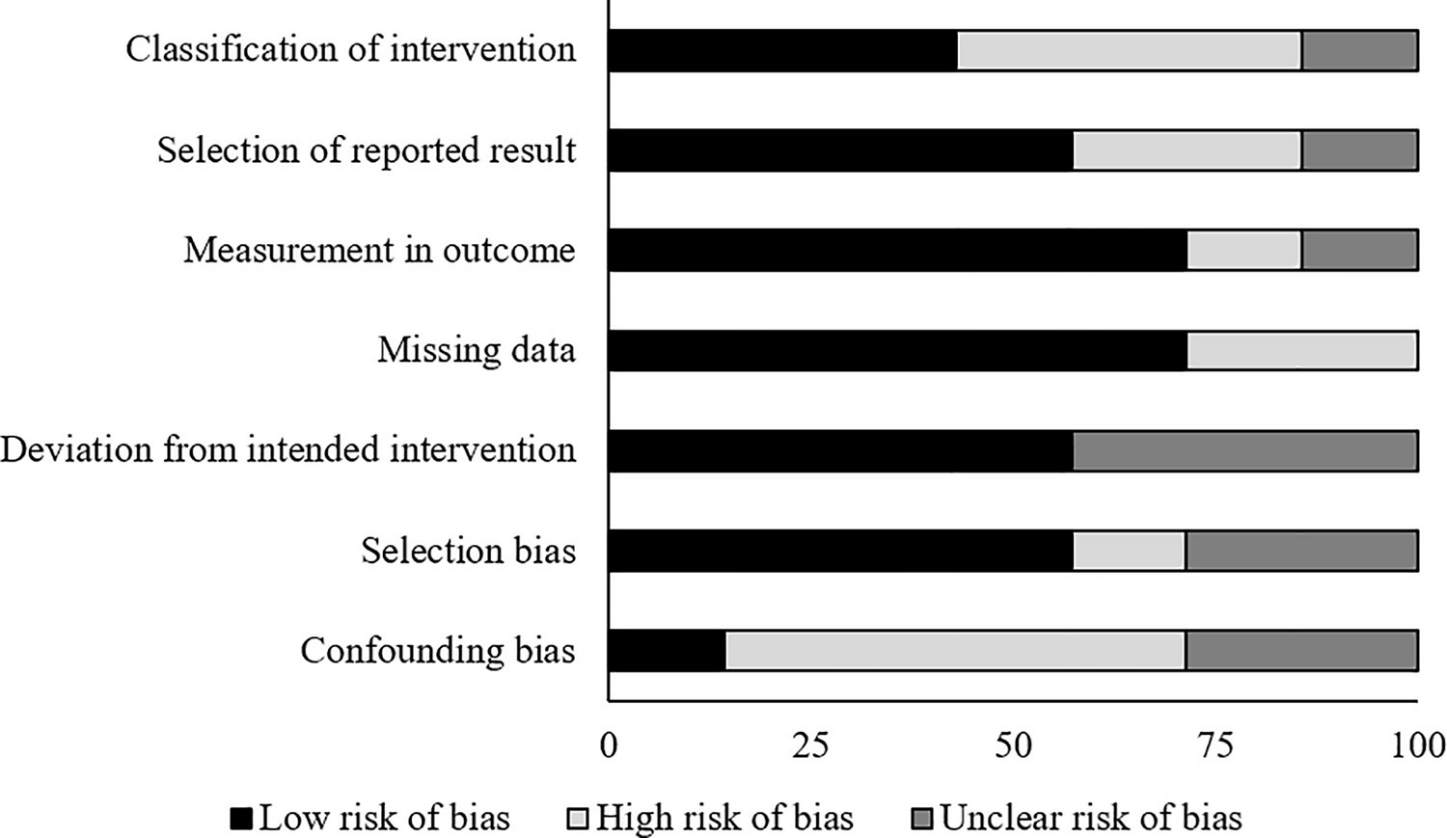
Illustrates the risk of bias (%) within studies according to Cochrane risk of bias assessment tool for controlled clinical trials.

The prevalence of risk of bias for the controlled clinical trials according to Cochrane’s risk of bias assessment tool for non-randomized controlled trials ROBINS-I has been demonstrated in Table 3. Here as well, the overall risk in the included studies is poor. The highest risk of bias was observed to be due to the lack of clarity in the confounding factors and classification of intervention Fig 3. Furthermore, a few studies refrained from explaining the measures they undertook to manage missing data and/or analyses for intention to treat analysis. A level of evidence of 2b was observed for all the included studies based on their experimental design.

**Table 3:** Illustrates the quality of the analyzed studies according to the Cochrane risk of bias assessment tool for non-randomized controlled trials ROBINS-I.

Study	Confounding bias	Selection bias	Deviation from intended intervention	Missing data	Measurement in outcome	Selection of reported result	Classification of intervention	Level of evidence
Josefsson et al. (1994)	**-**	**+**	**+**	**+**	**?**	**+**	**-**	2b
Chuckpaiwong et al. (2008)	**?**	**+**	**+**	**+**	**+**	**-**	**+**	2b
Ekstrand and van Dijk (2013)	**-**	**+**	**?**	**-**	**-**	**-**	**-**	2b
Kavanaugh et al. (1978)	**?**	**?**	**+**	**-**	**+**	**+**	**-**	2b
Park et al. (2017)	**+**	**+**	**?**	**+**	**+**	**+**	**?**	2b
Sokkar and Abdelkafy (2016)	**-**	**?**	**?**	**+**	**+**	**+**	**+**	2b
Lee et al. (2016)	**-**	**-**	**+**	**+**	**+**	**?**	**+**	2b

-: high risk of bias, +: low risk of bias,?: unclear risk of bias

### Publication bias

**Fig 4:**
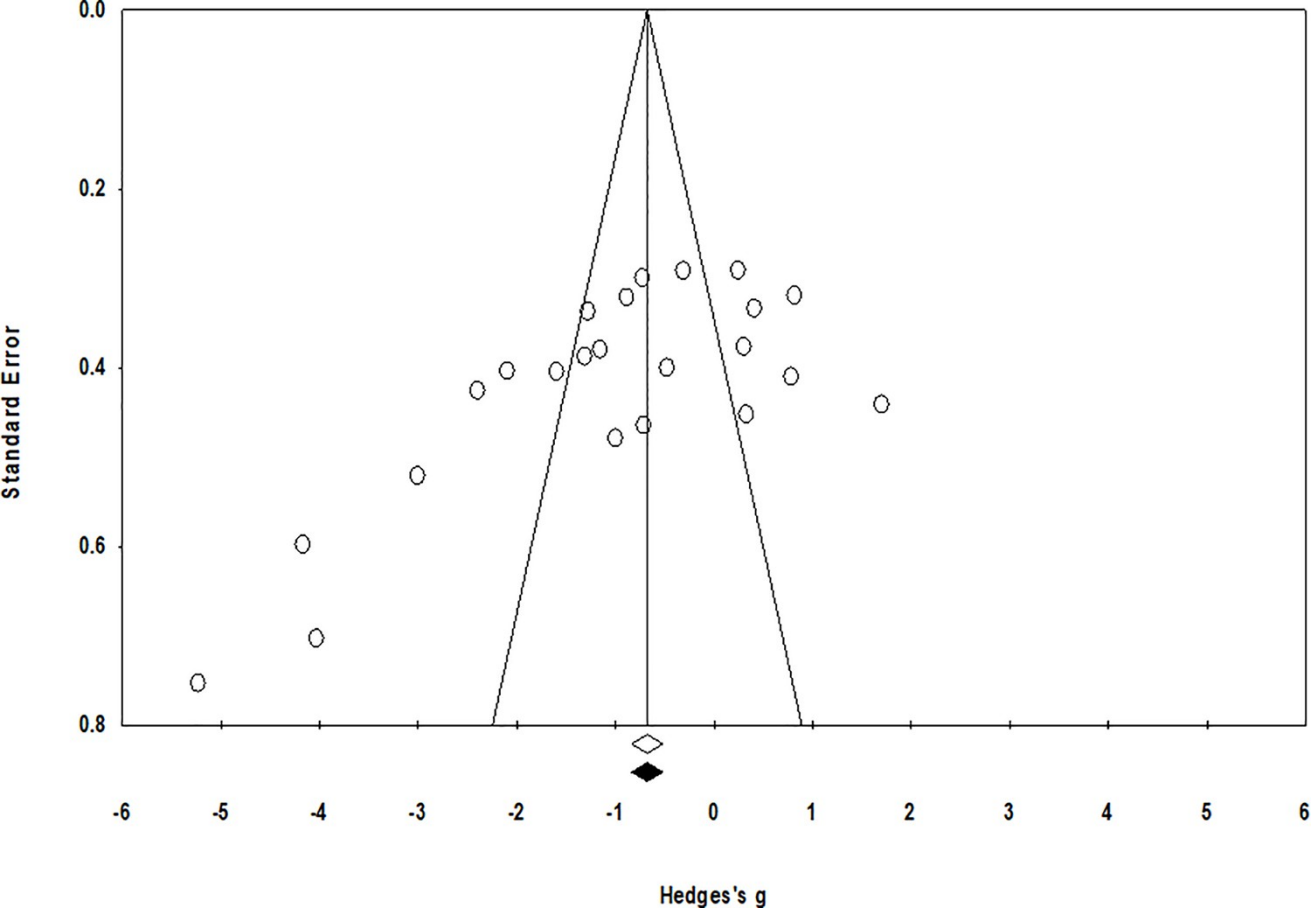
Illustrates the publication bias funnel plot by the Duval & Tweedie trim and fill procedure. Each of the analyzed effects is denoted by a circle in the plot. The boundaries of the plot mark the area where 95% of all the effects reside in case there were no publication biases. The vertical midline denotes the mean standardized effect of zero.

The trim and fill procedure identified no missing studies on the left or the right side of the mean effect (Fig 4). Further, according to the random-effect model, the point estimates and 95% confidence intervals for the evaluated parameters are -1.04 (-1.59 to -0.49). The trim and fill procedure report no changes in these values.

### Participant information

A total of 404 patients were evaluated in the studies included in this review. In the included studies four did not specify the gender of their sample [2, 47, 50, 52]. In the rest of seven studies [10, 48, 49, 51, 53–55], there were a total of 75 females and 170 males. Further, two studies did not define the sample distribution according to gender in the operated and non-operated groups [48, 53]. In the rest of the six studies [49, 49, 51, 54, 55], 98 (28F, 90M) patients were operated, whereas 81 (29F, 59M) patients were managed conservatively.

One of the included studies did not mention the age of the included sample [10]. Moreover, five studies reported the age of their sample as a range [2, 48, 51–53], and five reported the age as mean [47, 49, 50, 54, 55]. Six studies did not report the age distribution in their respective groups i.e. operated and non-operated [2, 48, 50–53]. In the studies that reported the age according to their respective groups, the mean age of the included participants was 31.7 ± 10.3 years for the group receiving operated management, and 31.4 ± 4.9 years for the group receiving non-operative management. Furthermore, the average duration of follow-up reported in the included studies was 1.73 ± 1.6 years. One study did not explicitly specify the duration of follow-up [51].

### Assessment

In the included studies, nine analyzed the influence of operative and non-operative interventions on the rate of non-union of the fifth metatarsal base fracture[2, 10, 47–52, 54]. Eight studies evaluated the mean duration of union [10, 48–50, 52–55], three evaluated the mean duration of return to sport [48, 50, 51], and two evaluated the mean duration of return to normal activities of daily living [10, 52]. Likewise, five studies each evaluated visual analog scale score [10, 49, 53–55], and American orthopedic & foot scale score [47, 49, 53–55].

#### Intervention

In seven of the included studies, internal fixation was done with an intramedullary screw [2, 10, 48, 50–52, 54]. Furthermore, one study each used bicortical cancellous screw [55], percutaneous screw [49], and Herbert type headless bolt [47]. One study did not report the type of screw they utilized to perform the fixation of the fractured metatarsal base [53].

### Meta-analysis reports

#### Rate of union

**Fig 5:**
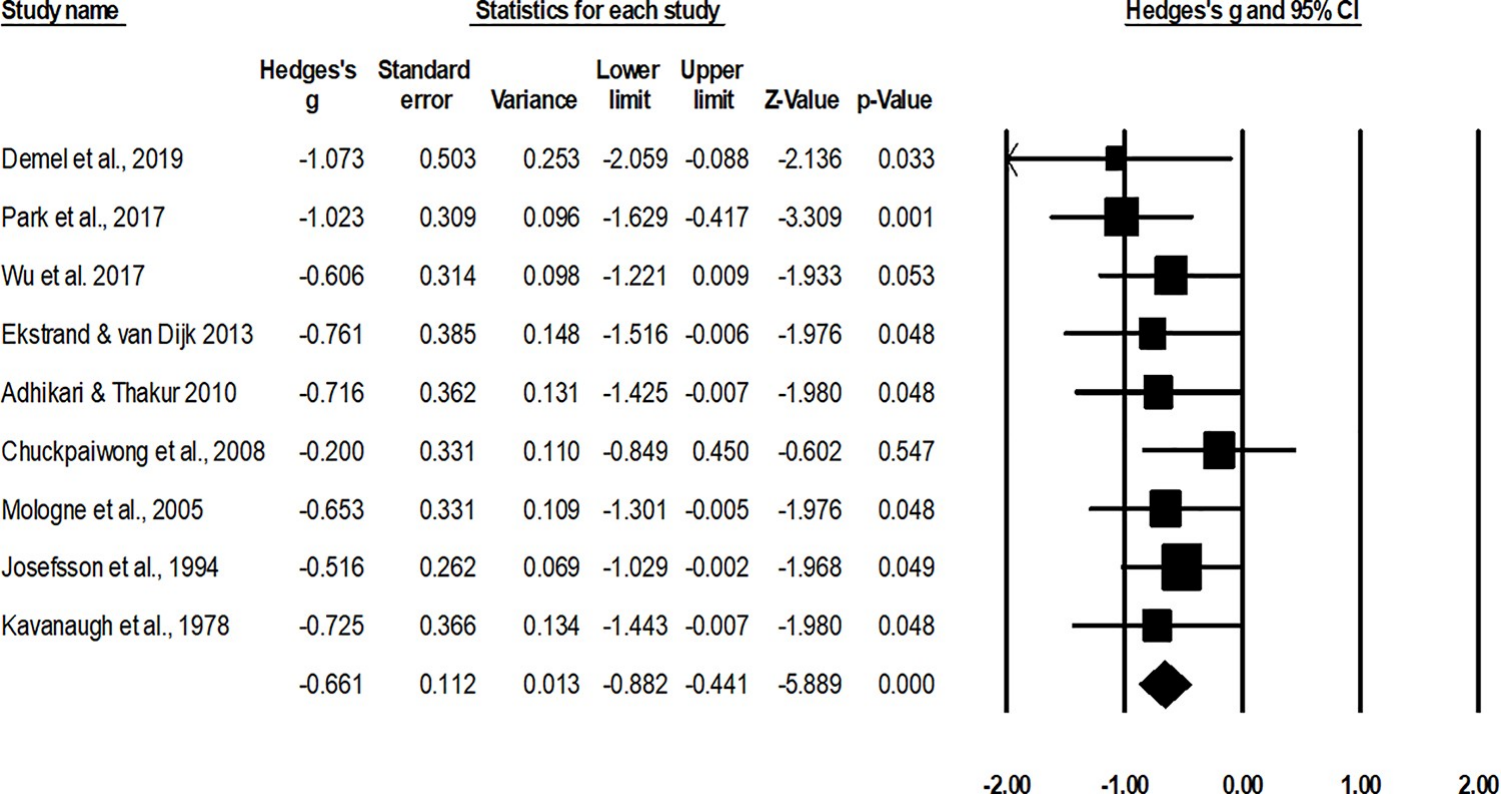
Illustrates the forest plot for studies comparing the rate of non-union between the groups receiving operated and non-operated management for the fifth metatarsal base fracture. Weighted effect size is presented as boxes, 95% C.I are presented as whiskers. A negative effect represents a reduced rate of non-union for the operated group; a positive effect represents a reduced rate of non-union for the non-operated group.

The assessment of mean healing duration was performed by nine studies [2, 10, 47–52, 54]. An across group, random-effect analysis (Fig 5) revealed a *medium* negative and significant effect of operative interventions to reduce the rate of non-union while managing fifth metatarsal base fracture as compared to non-operative conservative management (g: -0.66, 95% C.I: -0.88 to -0.44, p<0.01) with no heterogeneity (I^2^: 0%).

#### Duration of union

**Fig 6:**
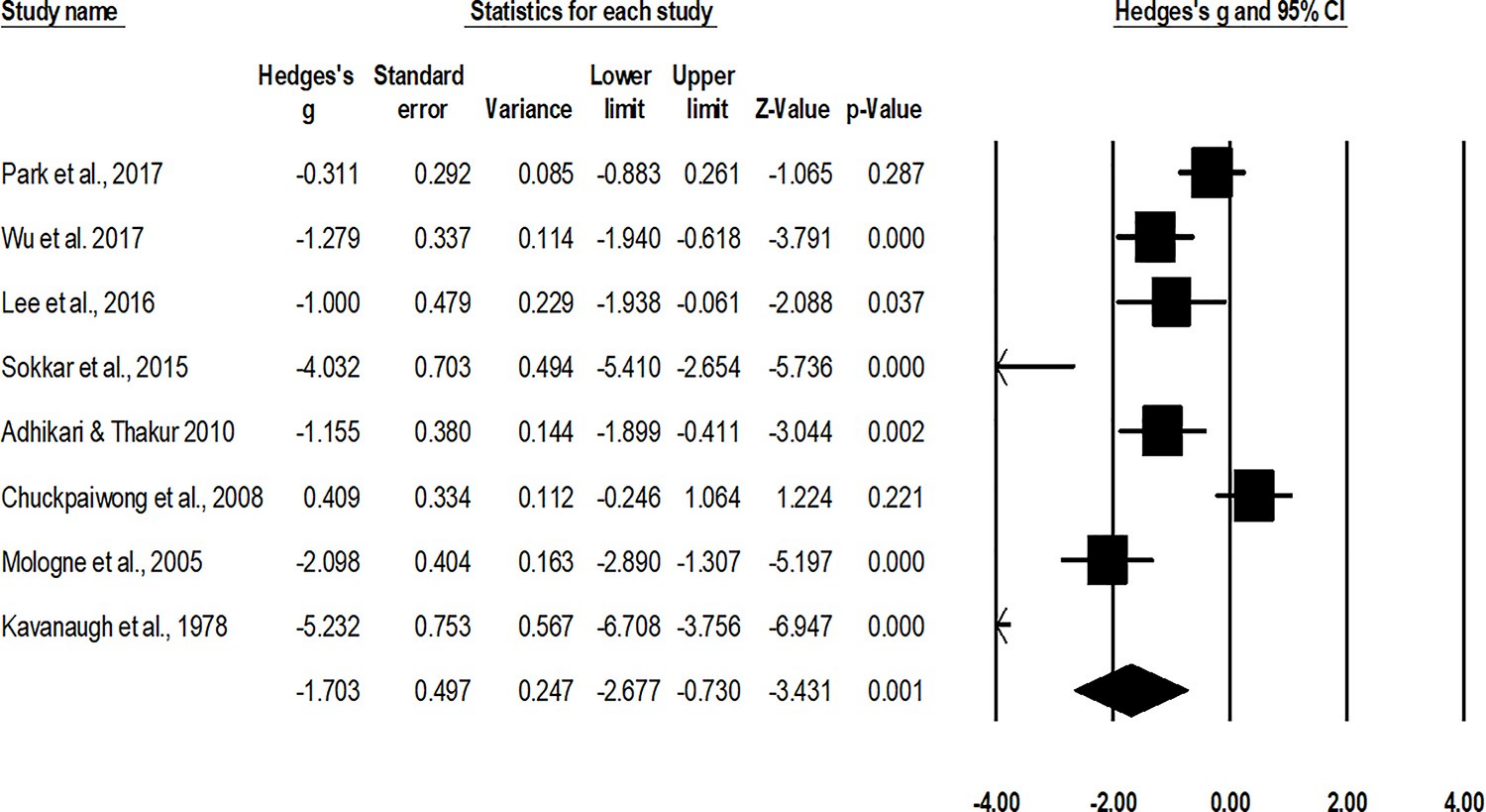
Illustrates the forest plot for studies comparing the mean duration of union between the groups receiving operated and non-operated management for the fifth metatarsal base fracture. Weighted effect size is presented as boxes, 95% C.I are presented as whiskers. A negative effect represents a reduced duration of union for the operated group; a positive effect represents a reduced duration of union for the non-operated group.

The assessment of duration of union was performed in eight studies [10, 48–50, 52–55]. An across group, random-effect analysis (Fig 6) revealed a *large* negative and significant effect of operative interventions to reduce the duration of union while managing fifth metatarsal base fracture as compared to non-operative conservative management (g: -1.7, 95% C.I: -2.6 to -0.73, p<0.01) with moderate heterogeneity (I^2^: 40.8%).

#### Duration of return to normal activity and sport

**Fig 7:**
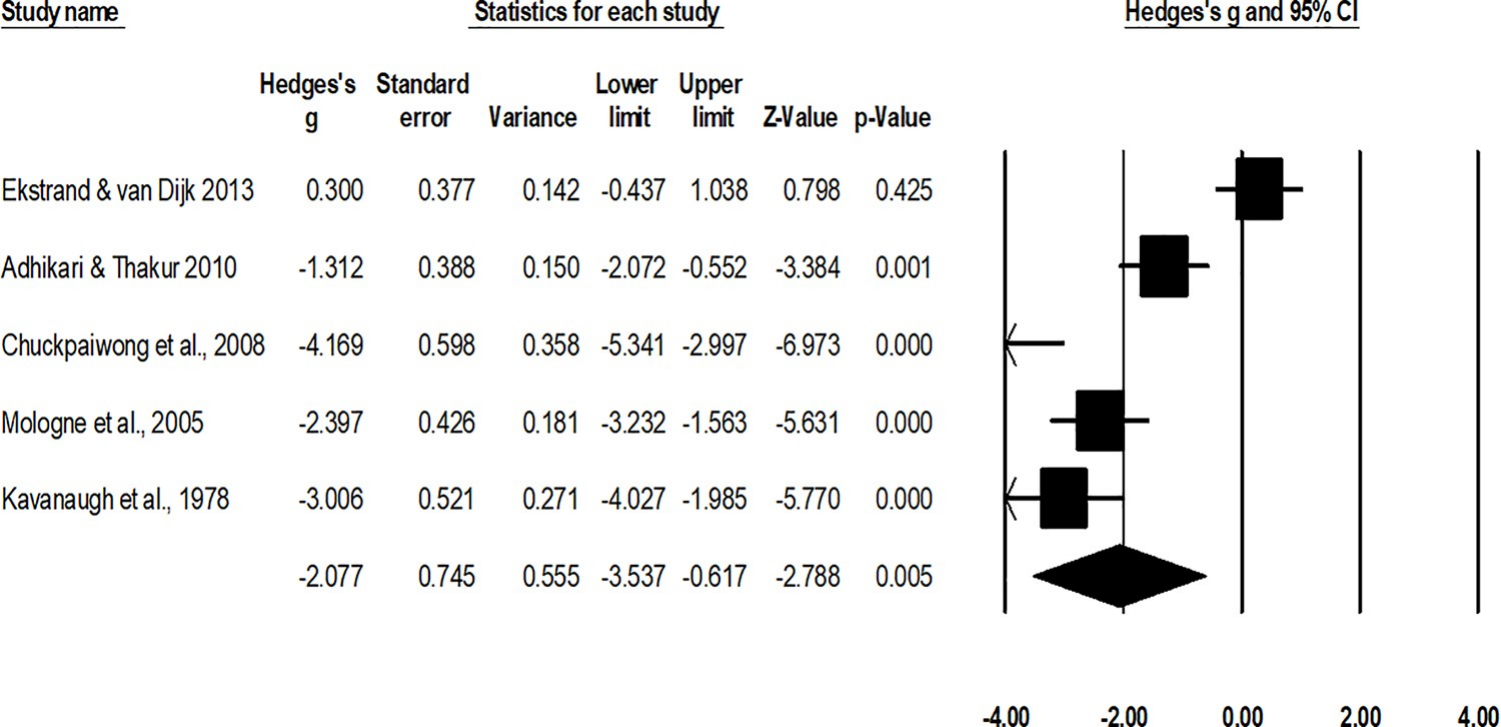
Illustrates the forest plot for studies comparing the mean duration of return to normal activity between the groups receiving operated and non-operated management for the fifth metatarsal base fracture. Weighted effect size is presented as boxes, 95% C.I are presented as whiskers. A negative effect represents a reduced duration of return to normal activity for the operated group; a positive effect represents a reduced duration of return to normal activity for the non-operated group.

The assessment of the mean duration of return to normal activity was done in two studies [10, 52], the assessment of mean duration of return to sport was done in three studies [48, 50, 51]. A combined, across group, random-effect analysis (Fig 7) revealed a *large* negative significant effect of operative interventions to reduce the duration of return to normal activity/sport after the management of fifth metatarsal base fracture as compared to non-operative conservative management (g: -2.07, 95% C.I: -3.5 to -0.61, p<0.01) with negligible heterogeneity (I^2^: 3.7%). Moreover, a subgroup analysis differentiating the effects of operative and non-operative interventions on return to normal activity and sport was performed.

**Fig 8:**
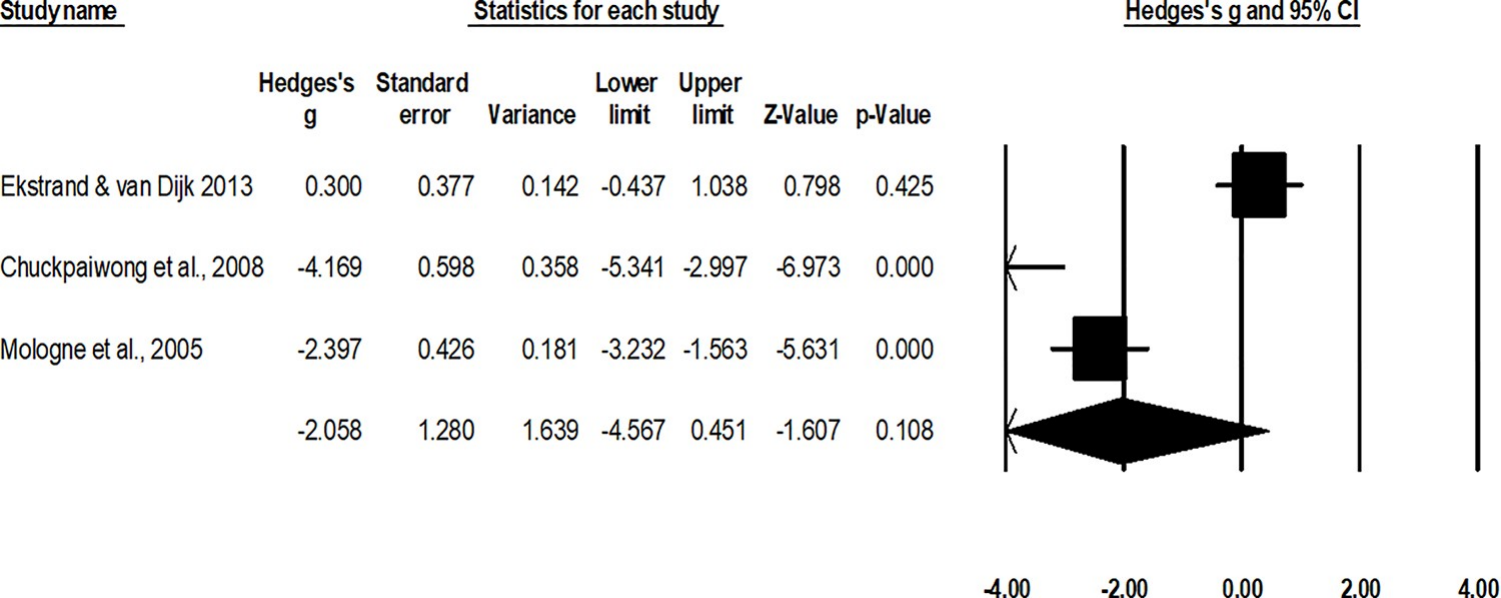
Illustrates the forest plot for studies comparing the mean duration of return to sport between the groups receiving operated and non-operated management for the fifth metatarsal base fracture. Weighted effect size is presented as boxes, 95% C.I are presented as whiskers. A negative effect represents a reduced duration of return to sport for the operated group; a positive effect represents a reduced duration of return to sport for the non-operated group.

For the analysis of duration of return to sport, the random-effect analysis (Fig 8) revealed a *large* negative non-significant effect of operative interventions to reduce the duration of return to normal sport after the management of fifth metatarsal base fracture as compared to non-operative conservative management (g: -2.05, 95% C.I: -4.5 to 0.45, p = 0.1) with negligible heterogeneity (I^2^: 2.7%).

**Fig 9:**
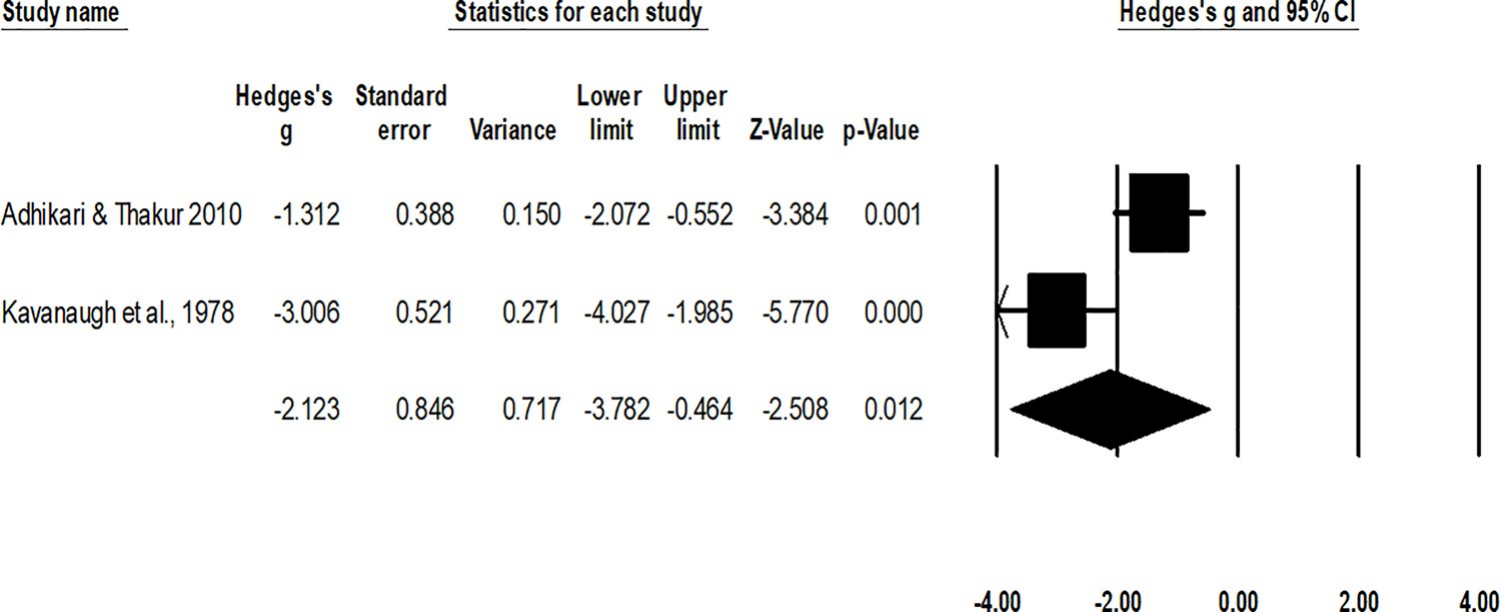
Illustrates the forest plot for studies comparing the mean duration of return to normal activity between the groups receiving operated and non-operated management for the fifth metatarsal base fracture. Weighted effect size is presented as boxes, 95% C.I are presented as whiskers. A negative effect represents a reduced duration of return to normal activity for the operated group; a positive effect represents a reduced duration of return to normal activity for the non-operated group.

For the analysis of duration of return to normal activity, the random-effect analysis (Fig 9) revealed a *large* negative significant effect of operative interventions to reduce the duration of return to normal sport after the management of fifth metatarsal base fracture as compared to non-operative conservative management (g: -2.12, 95% C.I: -3.7 to -0.46, p = 0.01) with no heterogeneity (I^2^: 0%).

#### Visual analog scale

**Fig 10:**
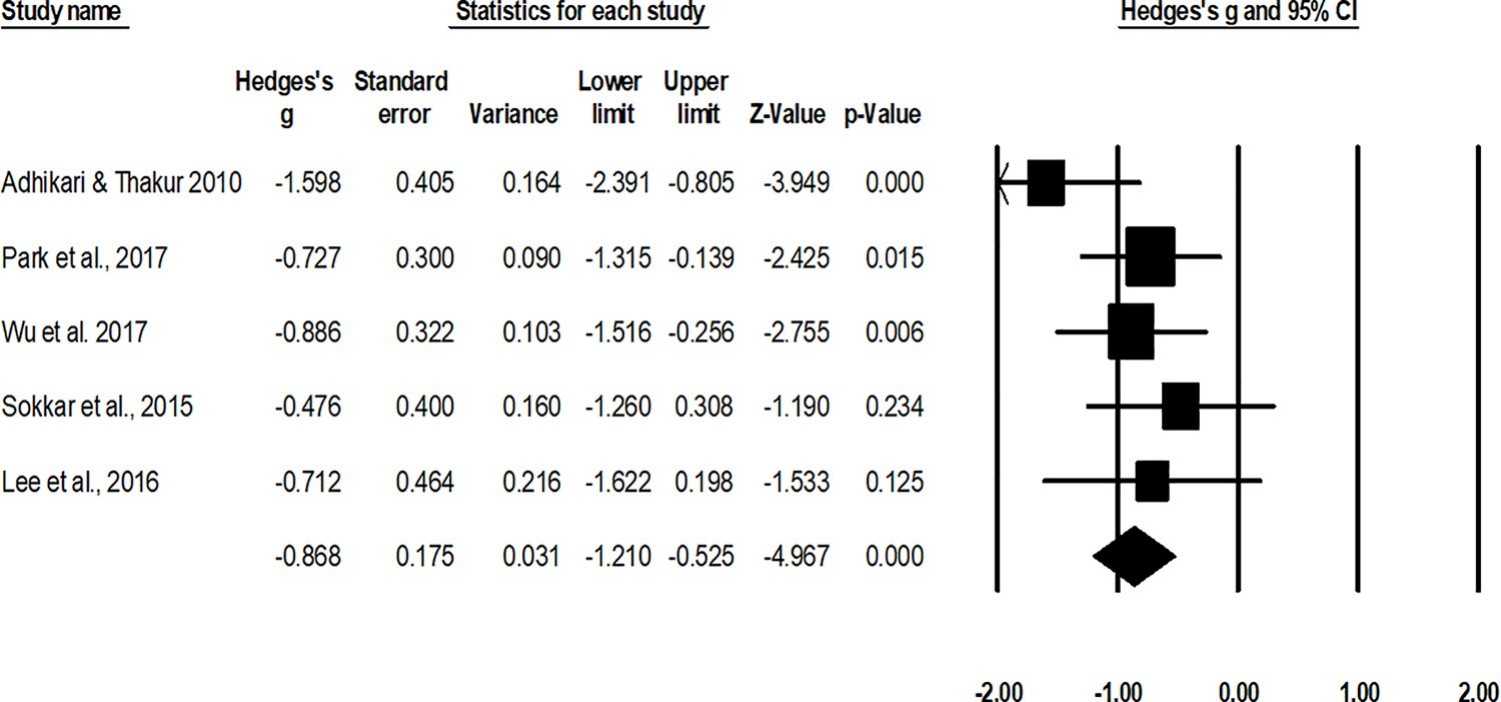
Illustrates the forest plot for studies comparing visual analog scale scores between the groups receiving operated and non-operated management for the fifth metatarsal base fracture. Weighted effect size is presented as boxes, 95% C.I are presented as whiskers. A negative effect represents a reduced visual analog scale score for the operated group; a positive effect represents a reduced visual analog scale score for the non-operated group.

The assessment of the perception of discomfort/pain via visual analog scale was done in five studies [10, 49, 53–55]. A combined, across group, random-effect analysis (Fig 10) revealed a *large* negative significant effect of operative interventions to reduce the perception of pain assessed via visual analog scale after the management of fifth metatarsal base fracture as compared to non-operative conservative management (g: -0.86, 95% C.I: -1.2 to -0.52, p<0.01) with negligible heterogeneity (I^2^: 1.6%).

#### American orthopedic foot & ankle scale

**Fig 11:**
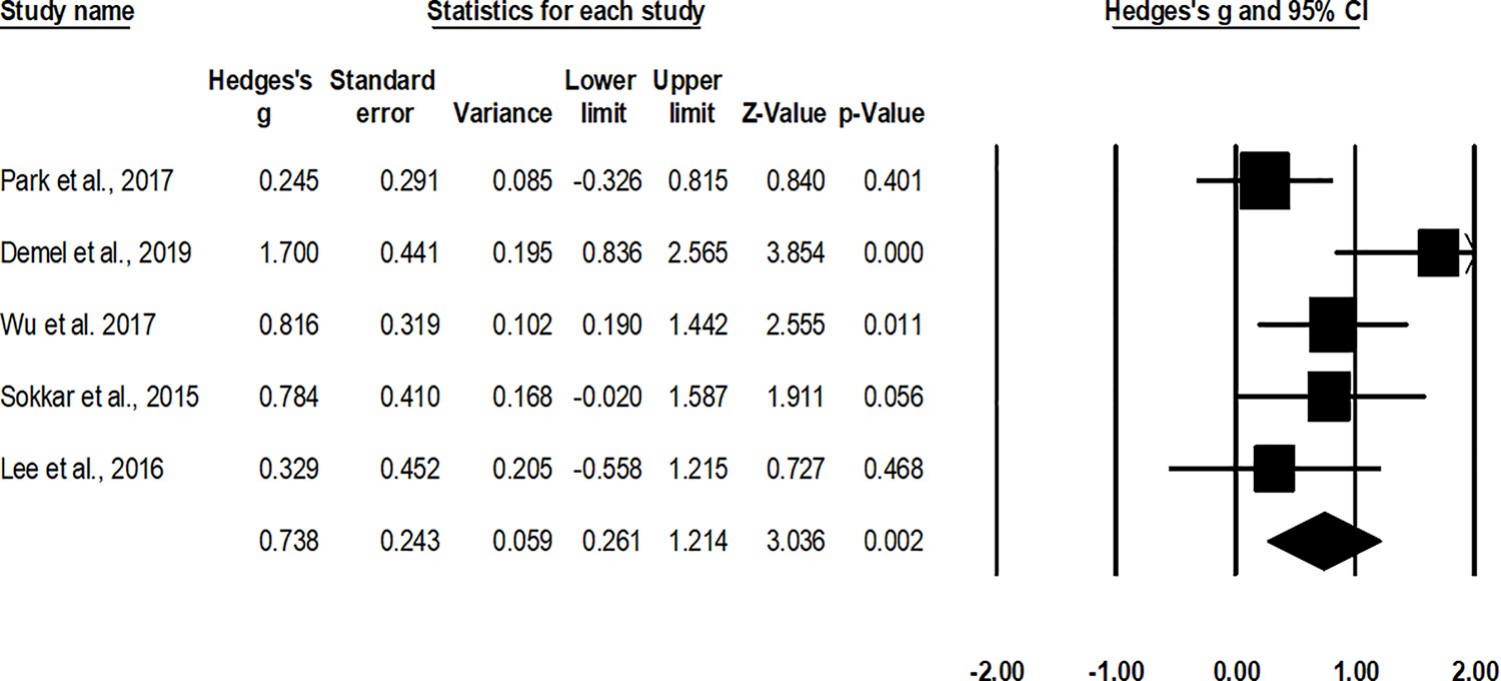
Illustrates the forest plot for studies comparing the American orthopedic foot & ankle scale scores between the groups receiving operated and non-operated management for the fifth metatarsal base fracture. Weighted effect size is presented as boxes, 95% C.I are presented as whiskers. A negative effect represents an increased American orthopedic foot & ankle scale score for the operated group; a positive effect represents an increased American orthopedic foot & ankle scale score for the non-operated group.

The assessment of the treatment outcome via the American orthopedic foot & ankle scale was done in five studies [47, 49, 53–55]. A combined, across group, random-effect analysis (Fig 11) revealed a *middle* positive significant effect of operative interventions to enhance the American orthopedic foot & ankle scale score after the management of fifth metatarsal base fracture as compared to non-operative conservative management (g: 0.73, 95% C.I: 0.26 to 1.2, p<0.01) with negligible heterogeneity (I^2^: 4.5%).

## Discussion

This review for the first time provides a comprehensive state of evidence concerning the outcome of the fifth metatarsal’s base fracture by comparing the effects of operative and non-operative interventions. We report beneficial effects of operative interventions for reducing the rate of non-union, duration of union, duration of return to normal activity, duration of return to sport and visual analog scale score as compared to non-operative interventions. Moreover, we report significantly enhanced treatment outcome as assessed with American orthopedic foot & ankle scale score for the operative interventions as compared to non-operative interventions.

The management of fracture at the base of the fifth metatarsal is a challenging avenue for an orthopedic because of its peculiar vasculature [12]. It has been documented that the presence of the watershed-region at the base of fifth metatarsal can substantially lower the success rate concerning the rate of union especially with non-operative interventions [56]. Due to this, the past decades have seen a substantial rise in the use of operative interventions for managing fractures at the base of the fifth metatarsal [48, 57]. The use of invasive interventions has primarily been favored because of their ability to provide a rigid fixation to the fractured surfaces while preserving the retrograde blood supply to the vascular insufficient areas [16, 34]. Porter (2018) mentioned that external fixation with a screw can on one hand efficiently resist the bending-moment at the fracture site. While on the other hand, it can also improve the vascularisation. The author mentions that the drilling procedure for the fixation could provide novel vascular access in the rather avascular part of the fracture site. This enhanced revascularization then could facilitate the healing process at the site thereby enhancing the overall prognostic outcome of the fracture. In agreement with these observations, this present systematic review too reports that operative interventions are able to significantly reduce the rate of union while enhancing the treatment-related outcomes. Wu et al. (2017), for example, compared the effectiveness of operative and non-operative interventions on 41 young adults, during a 1-year follow up. The authors reported that internal fixation of an avulsion fracture (2 to 3 mm displacement) allowed substantial enhancements in the clinical outcomes such as rate and duration of union as compared to the group administered immobilization with a plaster cast. Likewise, Mologne et al. (2005), Sokkar and Abdelkafy (2016) and Adhikari and Thakur (2010) also reported higher a success rate of operative interventions as compared to non-operative interventions. In this present meta-analysis, we confirm these findings statistically and report *medium* to *large* significant reduction of operative interventions on the rate on non-union (g: -0.66), and duration of union (-1.7) as compared to non-operative interventions. Moreover, the analysis reports enhancements in treatment-related outcome as assessed by the American orthopedic foot & ankle scale (0.73) when compared to non-operative interventions.

In addition, the use of operative interventions has been endorsed especially in sports settings [57]. Here, the use of operative interventions is preferred over its conservative counterpart because of its ability to promote an early weight-bearing on the affected extremity, eventually promoting an early return to the sports. Sokkar and Abdelkafy (2016), for instance, reported a significant reduction in the duration of return to sport in the operative group (7.1 weeks) as compared to the non-operative group (8.7 weeks). The authors hypothesized that the ability of the operative fixation to resist torsional strain at the fracture site, while promoting an equalized load dispersion could be the main reason due to which the early weight-bearing and return to sport is possible [58]. Similarly, Chuckpaiwong et al. (2008), Mologne et al. (2005) and Wu et al. (2017) too mentioned a significant reduction in the duration of return to sports in the groups where operative interventions were performed as compared to non-operative interventions. Park et al. (2017) further mentioned that a reduced level of bone resorption due to early weight-bearing could also be an additional reason behind this early yet robust reunion. This present meta-analysis again confirms these findings and reports *large* significant reduction in not only the duration of return to sport (-2.05) with operative interventions but also that of return to normal activities of daily living (-2.12). These findings, therefore, signify the importance of operative interventions to promote reunion in both sports and sedentary settings.

Finally, contrary to the reports in literature, which recommend the use of conservative management based on their ability to reduce the discomfort/pain suffered post-operatively, this present review reports reduction in the levels of discomfort/pain assessed via visual analog scale for the operative group [10, 49, 53–55]. Furthermore, the present meta-analysis also reports *large* effect size reduction in the levels of visual analog scale score (-0.86) for the operative group as compared to the non-operative group. Wu et al. (2017), proposed that the main underlying mechanism behind the onset of pain could possibly be an improper alignment of the fracture margins, which further can also promote abnormal pressure distribution on the plantar surfaces. The authors mentioned that in a comparison of cohorts getting operative and non-operative interventions, they observed higher rates of malunion within the non-operative group. Wu et al. (2017) further added that this higher rate of non-union could then possibly affect the mid-foot alignment or increase the risks for re-fracture, thereby causing an increased level of pain. Here, operative external fixations that provide a rigid approximation of the fracture margins while simultaneously reducing its impact on the arch-alignment could, therefore, be useful [20, 31].

Despite the novelty of this present meta-analysis, a few limitations persisted in this review. Firstly, registration of this systematic review was not performed in a prospective registry such as PROSPERO. This might raise questions concerning the validity of this review [59]. Secondly, we did not include unpublished papers in our systematic review. This was initially done to avoid biasing in the overall interpretation of the results; however, it does not rule out the possibility that some relevant results could be missing in the published literature. We recommend our reader to interpret the results considering this limitation. Thirdly, we did not evaluate the influence of operative and non-operative interventions based on the specific zones of fracture. These findings could have had an immense impact on developing efficient orthopedic care guidelines for an optimal choice of intervention to manage the fifth metatarsal’s base fracture. We strongly recommend future studies to address this issue by performing a meta regression-based analysis to compare the effects of the operative intervention on different zones of fracture. Fourthly, we also included one study that had incorporated patients with avulsion fracture at the fifth metatarsal’s base [49]. We understand that the peculiar vascularity of the fifth metatarsal’s base might not be affected cases of avulsion fracture, and that the inclusion of this study could have biased our interpretation. Therefore, we would recommend our reader to interpret the results carefully in the light of this factor.

Fifthly, we presume that the scarcity of statistical data in the included studies could have biased our interpretations concerning the influence of operative and non-operative interventions on the duration of return to normal activity. Here, evaluation of the duration of return to normal activity was performed only in two studies including a total of 28, 34 participants in the operative and non-operative groups, respectively. In this instance, the outcome of a *large* effect could suggest the possibility of a type II error, due to the small sample size [60]. Likewise, due to the limited availability of data, we were not able to carry out a cost-benefit analysis between the two types of interventions. We recommend future studies to address this paucity of data by evaluating the duration of return to normal activity and cost-benefit outcomes while sharing their descriptive statistics in open access data repositories. Evaluation of these parameters would be extremely beneficial for healthcare communities especially in low-and middle-income countries where morbidity associated with fifth metatarsal’s base fracture is the highest [61].

In conclusion, this systematic review and meta-analysis provide a 1b level of evidence to support the use of operative interventions to reduce the rate of non-union, duration of union, duration of return to sports, duration of return to normal activities of daily living, and visual analog scale score as compared to non-operative interventions. Besides, the operative interventions were also found to enhance the outcome of the treatment as assessed by the American orthopedic foot & ankle scale score to manage the fifth metatarsal’s base fracture as compared to non-operative interventions. The findings from the current meta-analyses can have widespread implications for developing best practice emergency orthopedic care guidelines for managing the fracture at the base of the fifth metatarsal.

## Supporting information

S1 File PRISMA checklist. (DOC)
